# Anthropometry relationship with duodenal histologic features of children with environmental enteric dysfunction: a multicenter cross-sectional study

**DOI:** 10.1016/j.ajcnut.2024.02.027

**Published:** 2024-09-17

**Authors:** Zehra Jamil, Kelley VanBuskirk, Monica Mweetwa, Samer Mouksassi, Gerald Smith, Tahmeed Ahmed, Kanta Chandwe, Donna M Denno, S Mohammad Fahim, Paul Kelly, Mustafa Mahfuz, Indika Mallawaarachchi, Chelsea Marie, Sean R Moore, William A Petri, S Asad Ali, EEDBI Consortium, Kumail Ahmed, Sheraz Ahmed, Md. Ashraful Alam, Beatrice Amadi, Rosemary Banda, Shareef Dars, Subhasish Das, Lee A. Denson, Md. Shabab Hossain, Aneeta Hotwani, Junaid Iqbal, Najeeha Talat Iqbal, Sadaf Jakhro, Furqan Kabir, Lydia Kazhila, Ta-Chiang Liu, Barbara J. Mann, Waheeda Memon, Christopher A Moskaluk, Abdul Khalique Qureshi, Shyam S Ragahavan, Masudur Rahman, Najeeb Rahman, Kamran Sadiq, Shafiqul Alam Sarker, Peter B. Sullivan, Phillip I. Tarr, Guillermo J. Tearney, Fayaz Umrani, Omer H. Yilmaz

**Affiliations:** 15 Department of Paediatrics and Child Health, Aga Khan University, Karachi, Pakistan; 16 International Centre for Diarrhoeal Disease Research, Bangladesh, Dhaka, Bangladesh; 17 Tropical Gastroenterology and Nutrition Group, University of Zambia School of Medicine, Lusaka, Zambia; 18 Division of Pediatric Gastroenterology, Hepatology, and Nutrition, Cincinnati Children's Hospital Medical Center, Cincinnati, OH, USA; 19 Department of Biological and Biomedical Sciences, Aga Khan University, Karachi, Pakistan; 20 Department of Pathology and Immunology, Washington University, St. Louis, MO, USA; 21 Department of Medicine, University of Virginia, Charlottesville, Virginia, USA; 22 Department of Pathology, University of Virginia School of Medicine, Charlottesville, VA, USA; 23 Department of Gastroenterology, Sheikh Russel National Gastroliver Institute and Hospital, Dhaka, Bangladesh; 24 Department of Paediatrics, Children's Hospital, University of Oxford, Oxford, UK; 25 Department of Pediatrics, Washington University School of Medicine, St. Louis, MO, USA; 26 Department of Pathology, Harvard Medical School, Boston, MA, USA; 27 Department of Pathology, Massachusetts General Hospital, Boston, MA, USA; 1 Department of Biological and Biomedical Sciences, Aga Khan University, Karachi, Pakistan; 2 Department of Global Health, University of Washington School of Public Health, Seattle, WA, United States; 3 Tropical Gastroenterology and Nutrition Group, University of Zambia School of Medicine, Lusaka, Zambia; 4 Certara, Princeton, NJ, United States; 5 Cytel, Vancouver, British Columbia, Canada; 6 Nutrition Research Division, International Centre for Diarrhoeal Disease Research, Bangladesh, Dhaka, Bangladesh; 7 Department of Pediatrics, University of Washington School of Medicine, Seattle, WA, United States; 8 Blizard Institute, Barts & The London School of Medicine, Queen Mary University of London, London, United Kingdom; 9 Department of Public Health Sciences, University of Virginia School of Medicine, Charlottesville, VA, United States; 10 Department of Medicine, University of Virginia School of Medicine, Charlottesville, VA, United States; 11 Department of Pediatrics, University of Virginia School of Medicine, Charlottesville, VA, United States; 12 Department University of Virginia, Charlottesville, VA, United States; 13 Department of Paediatrics and Child Health, Aga Khan University, Karachi, Pakistan

**Keywords:** environmental enteric dysfunction, anthropometry, duodenal histology, stunting, wasting

## Abstract

**Background:**

Environmental enteric dysfunction (EED) is a precursor of growth faltering in children living in impoverished conditions who are frequently exposed to environmental toxins and enteropathogens, leading to small bowel inflammatory, malabsorptive, and permeability derangements and low-grade chronic systemic inflammation.

**Objectives:**

We explored the association between anthropometrics and duodenal histologic features of EED among children from 3 lower middle-income country centers.

**Methods:**

In this cross-sectional study, Pakistani children (*n* = 63) with wasting, Bangladesh children (*n* = 116) with stunting or at risk for stunting (height-for-age *Z* score [HAZ] <−1 but ≥−2), and Zambian children (*n* = 108) with wasting or stunting received nutritional intervention. Children with anthropometric status refractory to intervention underwent endoscopy. Linear regression models included anthropometric around endoscopy, scores of histology parameters, and a global index score of EED—the total score percent-5 (TSP-5). Multivariable models were adjusted for center, age, sex, and histology slide quality.

**Results:**

Intersite variation was observed while exploring the association between anthropometrics and the TSP-5; for example, Pakistani children had the worst HAZ, yet their median TSP-5 score was lower than that of the other 2 centers. Even within each site, no overall pattern of higher TSP-5 score was observed with worsening HAZ. During univariate analysis, TSP-5 (coefficient: 0.01; 95% confidence interval [CI]: 0, 0.02), goblet cell depletion (coefficient: 0.22; 95% CI: 0.06, 0.37), and Paneth cell depletion (coefficient: 0.14; 95% CI: 0.01, 0.27) were associated with HAZ scores; however, they lost statistical significance in the multivariable models, with study center most strongly confounding the relationships seen in univariate models between anthropometry and histology.

**Conclusions:**

This study contributes a crucial negative finding that duodenal morphological features did not associate with anthropometric phenotypes; hence, anthropometric measurements may not be a suitable outcome measure for use in EED trials. Trial outcomes may need to be defined by combining the functional and structural elements of the gut to monitor EED.

## Introduction

Undernutrition affects 159 million children aged < 5 y, including 7% and 22% of children worldwide with wasting (defined as weight-for-height/length *Z* score [WHZ/WLZ] <−2) and stunted height growth (defined as height/length-for-age *Z* score [HAZ/LAZ]) <−2), respectively [1]. Stunting, and especially wasting, are associated with an increased risk of mortality [2]. Linear growth failure has also been associated with an increased risk of morbidity, reduced neurodevelopmental and cognitive function, and chronic noncommunicable diseases in adulthood [2,3]. The causes of stunting are multiple and include food insecurity, maternal factors, genetic influences, recurrent infections, chronic diseases, and environmental causes. Worldwide, the prevalence of stunting and wasting among children has declined, yet this reduction has been inconsistent and at an insufficient pace to meet international targets on this metric [1,4].

Preventive and therapeutic nutrition-specific interventions to address moderate-to-severe wasting have shown only modest impact [5]. Nutritional therapeutic interventions have shown effectiveness for wasting, whereas preventative nutrition interventions have shown mixed results, with prevention and treatment of stunting being particularly intractable [6,7]. Due to its multifactorial and refractory nature, efforts are underway to sharpen the understanding of causes of growth faltering and identify improved prevention and therapeutic interventions.

Environmental enteric dysfunction (EED) is a precursor of growth faltering among children living in impoverished conditions who are frequently exposed to environmental toxins and enteropathogens, leading to small bowel inflammatory, malabsorptive, and permeability derangements and low-grade chronic systemic inflammation. These processes lead to compromised growth, further exacerbated by inadequate nutritional intake [8,9]. The EED Biopsy Initiative (EEDBI) is a Consortium of studies aimed at gaining mechanistic insights into EED pathophysiology to improve diagnostic and severity grading opportunities and inform therapeutics, as the condition is primarily asymptomatic yet highly consequential [10]. The EEDBI Consortium studies utilized upper small bowel endoscopic biopsies and developed a gold standard rigorous histopathologic diagnostic scoring system [11]. In this report, we examined relationships between anthropometrics and duodenal histologic features among children with EED from 3 different lower middle-income countries.

## Methods

### Subjects and methods

This anthropometry study is a part of the larger EEDBI Consortium; detailed methodology, including enrollment criteria and study procedures, are reported elsewhere [10]. Pakistani children in the Study of Environmental Enteropathy and Malnutrition study (SEEM) were enrolled based on WHZ <−2 and received one sachet of Acha Mum (ready-to-use supplementary food) (520–550 kcal/d) for 2 mo. For children with WLZ <−3, 1–2 sachets were supplied as per the child’s weight (200 kcal/kg/d). In the Bangladesh Environmental Enteric Dysfunction study (BEED), children with stunting and “at risk for stunting” (LAZ <−1 but >−2) received an egg, milk, and micronutrients daily (520 kcal/d) for two mo. The Zambian children were recruited into the Biomarkers of Environmental Enteropathy in Children study (BEECH) based on weight-for-age *Z* score (WAZ), LAZ, or WLZ <−2, after which they received 100–200 g of High-Energy Protein Supplement (350–700 kcal), an egg (78 kcal), and micronutrients daily for 3–4 mo. Patients with refractory WLZ (SEEM), LAZ (BEED), or LAZ or WLZ (BEECH) to the intervention without identifiable causes of their poor growth identified on medical evaluation underwent gastroduodenoscopy. The number of children who responded positively after receiving intervention was 76/177 (42.9%) for SEEM, 107/950 (11.3%) for BEED, and 15/179 (8.4%) for BEECH. Comparison groups included children presenting for endoscopic small bowel biopsies for clinical indications at Cincinnati Children’s Hospital Medical Center and University of Virginia Medical Center in Charlottesville and not having any clinically identified gastrointestinal histopathology. Children from these centers with celiac disease diagnosed based on clinical presentation, serology, and histology were included as there were two comparison groups.

The histology scoring system and procedures are described in detail in a companion article in this series [11]. In brief, endoscopic mucosal pinch biopsies from the second or third duodenal segment were sectioned and stained with hematoxylin and eosin. Slide images uploaded to a telepathology platform were semiquantitatively scored across eight histologic parameters by 2 or 3 Consortium gastrointestinal pathologists (each parameter and score are defined in Supplementary Table 1). Slide consensus scores for each parameter were averaged across pathologist readings. Finally, a global index score—the total score percent-5 (TSP-5)—developed to identify EED and determine EED histopathologic severity was calculated from the five histology parameters most informative in identifying EED compared with a reference group of American children without a clinical or histopathologic diagnosis of gastrointestinal disorders: villus blunting, intraepithelial lymphocytes, goblet and Paneth cell depletion, and intramucosal Brunner’s glands. If >1 of these five parameters was not scorable because of technical issues, the TSP-5 was also considered nonscorable. When more than one slide image for an individual was available, average scores across slides were used [10,11].

Weight and recumbent length (per standard for children aged <2 y) were measured at regular intervals for the children in the 3 EED cohorts by trained research staff. All measurements were taken twice by one measurer with a maximum allowed difference of 50 g for weight and 7 mm for length, and the average of these 2 readings was noted. Weights were measured using a Tanita (1584) scale in kilograms to the nearest 5 g ≤7.5 kg and the nearest 10 g ≤20 kg, and the machine was calibrated on a daily basis. Length was measured using an infantometer with the child’s head against the fixed headboard and the heel touching the moveable footboard. The length was noted to the last complete unit in mm. Weight and standing height (per standard for children aged >2 y) were abstracted from clinical records for the United States cohorts. WAZ and HAZ or LAZ (hereinafter referred to as HAZ) were calculated for all children using WHO Anthro software. Similarly, WHZ or WLZ (hereinafter referred to as WHZ) for children aged <5 y were calculated per WHO growth standard parameters. Anthropometric indices closest to the time of biopsy were used for this analysis; for clinical relevance, measurements were restricted to those collected within ±60 d of biopsy.

Signed informed consent from legal guardians was obtained, and all study centers obtained approvals from their review boards (Ethical approval was obtained by Ethics Review Committee (ERC) from AKU (3836-Ped- ERC-15), icddr,b ERC (PR-16007), University of Zambia Biomedical Research Ethics Committee (006-02-16) and National Health Research Authority (MH/101/23/10/1), UVA Institutional Review Board (19466), CCHMC Institutional Review Board (2016-0387). Exemption was received from the University of Washington Institutional Review Board (IRB) (STUDY00013442) and the Washington University IRB (201801207)); details on these procedures can be found elsewhere in this supplemental issue [10].

### Data analysis

Summary statistics of anthropometry and histology data were calculated. To determine whether histology score(s) predict anthropometry, linear regression was used with histology scores as predictors and anthropometry *Z* scores modeled as continuous variables as outcomes. Study center, sex, age, and 3 histology slide quality measures (staining quality, tissue dry/crush artifact, and tissue orientation) were included in multivariable models as possible confounders. The residuals in regression models did not violate normality assumptions. Statistical significance was defined as *P* < 0.05: all reported *P* values are 2-sided. Analyses were performed with R (version 4.1.3).

## Results

Two hundred ninety-one children from the 3 EED cohorts and 66 children from the United States cohorts underwent endoscopy [8]. Four children in the BEED cohort and one in the United States nondiagnostic cohort did not have anthropometry measurements within 60 d of their endoscopic procedure and were therefore excluded from this analysis; 352 children were included in this analysis (Table 1) and assigned as EED (*n* = 287), nondiagnostic comparison group (*n* = 43), and celiac comparison group (*n* = 22). Further characteristics of study participants are described elsewhere in a companion manuscript in this supplemental issue [8]. Consistent with their recruitment criteria, the Zambian cohort had the worst HAZ scores, and the Pakistani cohort had the worst WLZ and WAZ scores, whereas the Bangladeshi cohort was least undernourished (Table 2). More than half (*n* = 65, 56%) of the Bangladeshi cohort was stunted whereas the remainder had HAZ scores between <−1 and ≥−2; 11 (9.5%) children in this cohort were wasted (Supplementary Table 2). The vast majority of children in the Zambian cohort (*n* = 106, 98%) was stunted, although only 5 (5%) were wasted. The Pakistani cohort had the highest proportion of children who were both stunted and wasted (*n* = 32, 51%), and overall, 52 (83%) were stunted and 38 (60%) wasted. Median HAZ, WHZ, and WAZ were −2.9, −2.2, and −1.1 across the 3 EED centers.

**TABLE 1:** Descriptive statistics of the study participants by study center and disease category

	SEEM *n* = 63	BEECH *n* = 108	BEED *n* = 116	US Nondiagnostic *n* = 43	US Celiac *n* = 22
Sex, % female	30.2%	50.9%	57.8%	46.5%	54.5%
Age (y) at the time of biopsy, median (IQR)	1.7 (1.3, 1.8)	1.6 (1.3, 1.8)	1.6 (1.4, 1.7)	6.7 (5.1, 9.4)	7.1 (5.2, 10.0)
Age (y) at the time of anthropometry, median (IQR)	1.7 (1.3, 1.8)	1.6 (1.3, 1.8)	1.5 (1.4, 1.7)	6.7 (5.1, 9.4)	7.1 (5.2, 10.0)
Difference between biopsy and nearest anthropometry (d), median (IQR)	11.0 (5.0, 14.0)	1.0 (1.0, 1.0)	15.0 (12.0, 22.0)	1.0 (1.0, 1.0)	1.0 (1.0, 1.0)

Abbreviations: BEECH, Biomarkers of Environmental and Enteropathy in Children; BEED, Bangladesh Environmental Enteric Dysfunction; IQR, interquartile range; SEEM, Study of Environmental Enteropathy and Malnutrition.

**TABLE 2:** Anthropometry summary statistics by study center and disease category

Statistic	SEEM	BEECH	BEED	EED sites combined	US Nondiagnostic	US Celiac
HAZ						
Median	−3.2	−3.3	−2.1	−2.9	0.1	0.1
Q25	−3.7	−3.9	−2.8	−3.5	−0.6	−0.7
Q75	−2.4	−2.8	−1.6	−2.1	1	1.1
*n*	63	108	116	287	43	22
WAZ						
median	−3.1	−2.3	−1.7	−2.2	0.1	0.1
q25	−3.6	−2.7	−2.3	−2.8	−0.9	−0.7
q75	−2.6	−1.8	−1.3	−1.6	1.2	0.4
*n*	63	108	116	287	43	22
WHZ^1^						
Median	−2.2	−0.8	−1.0	−1.1	−0.6	−0.6
Q25	−2.8	−1.3	−1.4	−1.8	−1.0	−0.6
Q75	−1.8	−0.3	−0.4	−0.5	0	0.2
*n*	63	108	116	287	10	5

Abbreviations: BEECH, Biomarkers of Environmental and Enteropathy in Children; BEED, Bangladesh Environmental Enteric Dysfunction; CCHMC, Cincinnati Children’s Hospital Medical Center; EED, environmental enteric dysfunction; HAZ, height-for-age *Z* score; Q25, 25% quantile; Q75, 75% quantile; SEEM, Study of Environmental Enteropathy and Malnutrition; WAZ, weight-for-age *Z* score; WHZ, weight-for-height *Z* score; UVA, University of Virginia.

1 WHZ could only be calculated for children <5 y. All EED cohorts included children <5 y. CCHMC celiac cohort included 4, UVA celiac included 1, CCHMC nondiagnostic included 10, and UVA nondiagnostic included 0. Nondiagnostic and celiac cases are from American sites.

Scores for all histologic parameters by center and disease are summarized in Supplementary Table 3. The summary statistics for the EED summary index score—the TSP-5—is displayed by 4 HAZ categories corresponding to severe stunting, moderate stunting, mild stunting (HAZ < −1 but > −2), and no stunting (HAZ > −1) in Table 3. Although more than half of the Pakistani children fell in the worst HAZ category, their median TSP-5 score was better (lower) than the other 2 EED cohorts. Within any particular center, no overall pattern of higher TSP-5 scores was observed with the worsening of HAZ scores. We also examined the TSP-5 by WAZ and WHZ groups and similarly did not identify any histology score–anthropometry group patterns (Supplementary Table 4).

**TABLE 3:** Distribution of total score percent-5 (possible range 0–100%) of study participants segregated into 4 categories based on HAZ scores

HAZ categories	Statistic	SEEM (%)	BEECH (%)	BEED (%)	EED all sites combined (%)	Normal (%)	Celiac (%)
(−6.6 to <−3)	Median	47.2	53.5	60.3	52.2	0	0
	Q25	35.7	46.8	49.3	44.4	0	0
	Q75	53.2	58.3	69.4	58.3	0	0
	*n*	35	71	16	122	0	0
(−3 to <−2)	Median	41.6	57	57.5	55.6	16.1	0
	Q25	36.1	49.4	49.9	44.4	16.1	0
	Q75	52.1	63.3	64.6	63.3	16.1	0
	*n*	17	31	30	78	1	0
(−2 to <−1)	Median	45.9	43.6	57.8	49.8	21.1	36.1
	Q25	41.3	41.8	47.2	44.9	10.2	32
	Q75	49	45.4	68.2	65.2	22.2	37.5
	*n*	8	2	32	42	7	3
(−1 to 6)	Median	44.1	0	0	44.1	9.4	47.2
	Q25	42.5	0	0	42.5	7.15	31.9
	Q75	53.4	0	0	53.4	17.3	51.4
	*n*	3	0	0	3	35	19

Nonscorable included in the TSP-5: BEECH = 4 (3.7%), BEED = 38 (32.8%). Nondiagnosed and Celiac cases are from American sites.

Abbreviations: BEECH, Biomarkers of Environmental and Enteropathy in Children; BEED, Bangladesh Environmental Enteric Dysfunction; EED, environmental enteric dysfunction; HAZ, height-for-age *Z* score; Q25, 25% quantile; Q75, 75% quantile; SEEM, Study of Environmental Enteropathy and Malnutrition.

**FIGURE 1:**
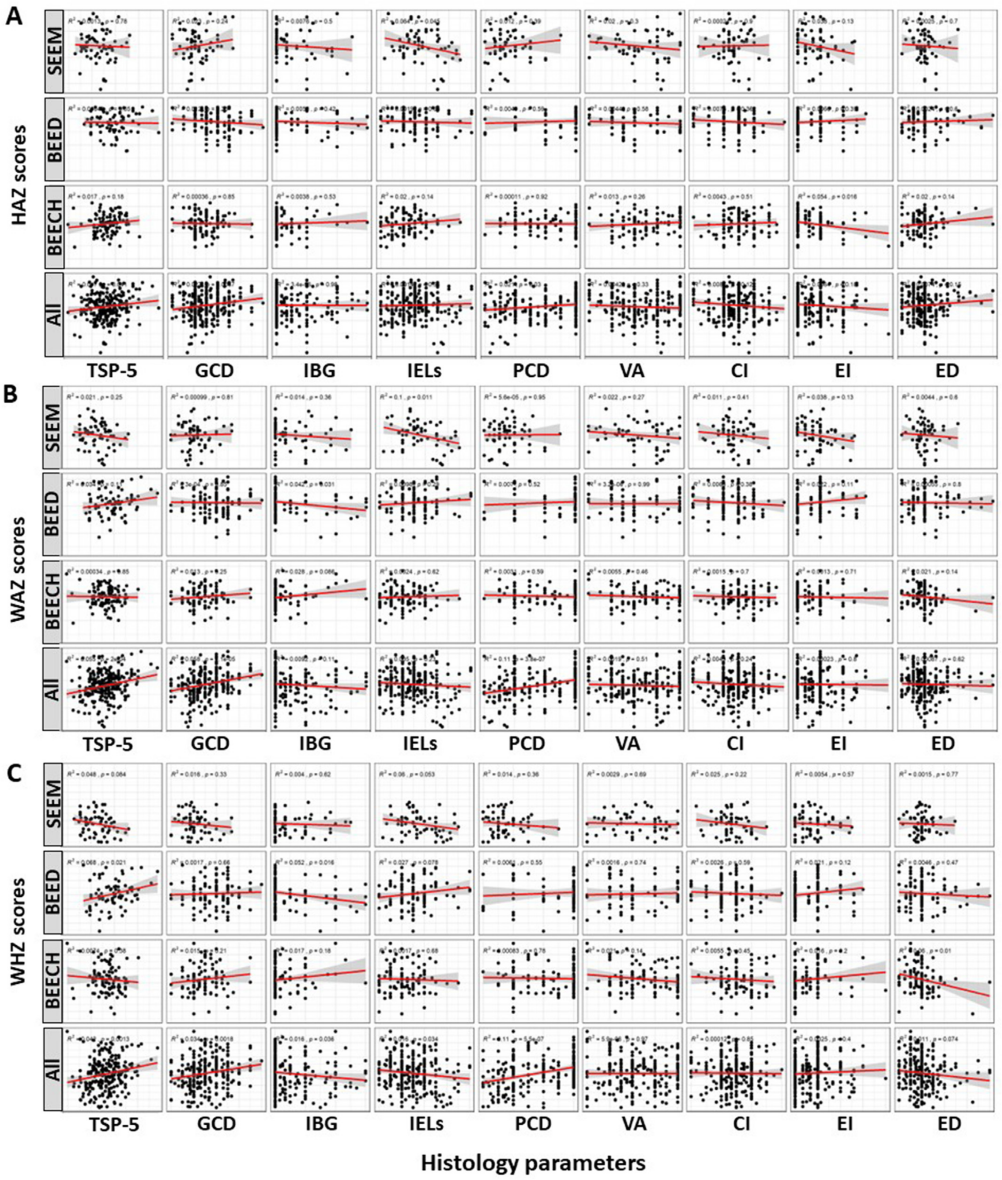
Univariate linear regression models showing association between histology parameters and anthropometry. (A) Height-for-age Z score (HAZ), (B) weight-for-age Z score (WAZ), and (C) weight-for-height Z score (WHZ) at each site (SEEM, Study of Environmental Enteropathy [Pakistan]; BEECH, Biomarkers of Environmental and Enteropathy in Children [Zambia]; BEED, Bangladesh Environmental Enteric Dysfunction) and all EED sites combined. CI, chronic inflammation; ED, epithelial detachment; EED, environmental enteric dysfunction; EI, enterocyte injury; GCD, goblet cell depletion; IBG, intramucosal Brunner’s gland; PCD, Paneth cell depletion; TSP-5, total score percent-top 5; VA, villus architecture,

Next, we performed univariate and multivariable regression to assess if histology parameters are associated with anthropometry (modeled as continuous variables) among children with EED (Table 4, Figure 1). Multiple histology parameter–*Z* score associations were seen in univariate analyses; however, they lost statistical significance in the multivariable models, with study center most strongly confounding the relationships between anthropometry and histology in most of the multivariable models.

**TABLE 4:** Linear regression results for anthropometry and histology scores

Anthropometry parameter	Histology variables	*n*	Univariable model		Multivariable model	
			Coefficient	95% CI	Coefficient	95% CI
WHZ	TSP-5	245	0.02	(0.01, 0.03)	0	(−0.01, 0.01)
	Goblet cell depletion	285	0.26	(0.1, 0.42)	0.06	(−0.09, 0.21)
	Intraepithelial lymphocytes	285	−0.16	(−0.31, −0.01)	0	(−0.14, 0.13)
	Intramucosal Brunner’s glands	282	−0.19	(−0.37, −0.01)	−0.1	(−0.25, 0.05)
	Paneth cell depletion	220	0.36	(0.22, 0.5)	−0.02	(−0.18, 0.13)
	Villus architecture	229	0	(−0.13, 0.12)	−0.04	(−0.15, 0.06)
	Chronic inflammation	283	−0.02	(−0.25, 0.2)	−0.15	(−0.34, 0.04)
	Enterocyte injury	286	0.14	(−0.19, 0.47)	0.19	(−0.09, 0.46)
	Epithelial detachment	287	−0.18	(−0.37, 0.02)	−0.16	(−0.33, 0)
WAZ	TSP-5	245	0.02	(0.01, 0.03)	0	(−0.01, 0.01)
	Goblet cell depletion	285	0.31	(0.17, 0.46)	0.02	(−0.12, 0.16)
	Intraepithelial lymphocytes	285	−0.08	(−0.22, 0.05)	−0.04	(−0.17, 0.08)
	Intramucosal Brunner’s glands	282	−0.13	(−0.3, 0.03)	−0.09	(−0.23, 0.04)
	Paneth cell depletion	220	0.33	(0.2, 0.45)	−0.01	(−0.15, 0.13)
	Villus architecture	229	−0.04	(−0.15, 0.07)	−0.05	(−0.14, 0.05)
	Chronic inflammation	283	−0.12	(−0.33, 0.08)	−0.14	(−0.31, 0.04)
	Enterocyte injury	286	−0.04	(−0.34, 0.27)	−0.03	(−0.29, 0.22)
	Epithelial detachment	287	−0.05	(−0.23, 0.14)	−0.07	(−0.22, 0.08)
HAZ	TSP-5	245	0.01	(0, 0.02)	0	(−0.01, 0.01)
	Goblet cell depletion	285	0.22	(0.06, 0.37)	−0.05	(−0.2, 0.1)
	Intraepithelial lymphocytes	285	0.04	(−0.1, 0.19)	−0.09	(−0.21, 0.04)
	Intramucosal Brunner’s glands	282	0	(−0.17, 0.17)	−0.04	(−0.18, 0.11)
	Paneth cell depletion	220	0.14	(0.01, 0.27)	0.02	(−0.13, 0.16)
	Villus architecture	229	−0.06	(−0.17, 0.06)	−0.03	(−0.13, 0.08)
	Chronic inflammation	283	−0.17	(−0.38, 0.04)	−0.04	(−0.23, 0.14)
	Enterocyte injury	286	−0.22	(−0.53, 0.1)	−0.28	(−0.55, −0.01)
	Epithelial detachment	287	0.14	(−0.05, 0.33)	0.08	(−0.08, 0.25)

All associations in univariate models with *P* < 0.05 were further explored in multivariable regressions adjusted for study center, sex, age, and 3 slide quality parameters.

Abbreviations: CI, confidence interval; HAZ, height-for-age *Z* score; TSP, total score percent; WAZ, weight-for-age *Z* score; WHZ, weight-for-height Z score.

## Discussion

This study explored the association between anthropometrics and EED duodenal histologic features. After adjusting for potential confounders, no association was seen between HAZ, WAZ, or WHZ and histology features or the summary EED index score—the TSP-5. Our study contributes a crucial negative finding that morphologic features in the duodenal tissue have no association with the anthropometric phenotypes; hence, growth measurements may not be a suitable outcome to monitor EED when EED is measured in terms of the histologic features that we used.

Our multicenter data exploring the relationship between gut histology and anthropometrics in 2 South Asian and 1 African cohort is an effort to advance the field of EED. Archival data from 2 hospitals in United States were included to overcome the challenge of the nonavailability of healthy gut histology data from the same centers. The EED histology scoring system that was employed in this analysis has been validated to distinguish biopsies of children with EED from those of children in United States with no gastrointestinal diagnosis [11], suggesting the relevance of gut histology even in the absence of correlation with growth parameters. Furthermore, the histology scoring system results in a range of score severity and variation in individual histology parameters among children with EED. Our study cohort included diverse undernutrition phenotypes from the 3 centers, spanning from children with mild stunting to those who were both wasted and severely stunted. However, only children with growth refractory to nutritional intervention underwent exploratory upper gastrointestinal endoscopy; hence, the range of *Z* scores was lower and narrower by study design compared with what would be expected among a general cohort of children with EED. This could have mitigated our attempts to associate histology with anthropometry despite prior evidence that EED encompasses a broad range of functional components including gut inflammation with or without systemic inflammation, which has been linked to ponderal and linear growth restriction in young children [12,13].

Optimal growth depends on multiple factors, including genetics and environmental conditions and prenatal conditions. In EED-endemic settings, recurrent infections, food insecurity, and micronutrient deficiencies are significant contributors to poor growth [14]. To overcome these factors, cases refractory to nutritional intervention were selected for upper gastrointestinal endoscopy. However, other causes could have also been contributing to the anthropometric status of the participants in addition to EED. Nevertheless, further studies are required to explore other factors, such as gut microbiome, that may hinder response to various interventions in addition to gut histology. Recently, in a Bangladeshi study comprising 110 young children with stunting refractory to nutritional intervention, bacterial strains belonging to 14 taxa were identified in the duodenal microbiome that showed a negative correlation with linear growth [15].

The limitations of this study include differences in the recruitment criteria and nutritional treatment provided prior to endoscopy across the 3 study sites that we attempted to correct by adjusting for study center in the analysis. Therefore, future studies might benefit from recruiting children with similar anthropometric criteria. Moreover, this study does not include functional assessments for EED, and the anthropometry was only assessed by static Z scores and not by longitudinal growth monitoring over time.

In conclusion, although the profile and severity of EED gut histological abnormalities showed some distinct differences across 3 EED-endemic settings, the histologic severity was not associated with anthropometry. This could be due to different cohort phenotypes and enrollment criteria or suggests more than one histologic pathway with geographic variation. This could also be because EED might be one of the factors contributing to the poor anthropometry but not the only factor singularly responsible for it. It could also be because EED may need to be defined by combining both functional and structural elements of the gut. The structural lens alone may not be adequate to classify and categorize EED. Future studies should, with more uniform enrollment criteria, evaluate relationships between anthropometry and histology and functional assessments of gut function.

## Author contributions

The authors’ responsibilities were as follows – SAA, KC, CM, TA, MMa, PK, SRM, WAP: designed research; SAA, KC, SMF, IM, CM, TA, MMa, PK, SRM, WAP, ZJ: conducted research; SM, GS, KV, MMw: analyzed data; SAA, ZJ: wrote the paper; SAA: had primary responsibility for final content; DMD: provided data management; KV, DMD: provided data interpretation; DMD: provided substantial manuscript editing; and all authors: read and approved the final manuscript.

### Conflict of interest

The authors report no conflicts of interest.

## Funding

The EEDBI Consortium was funded by the following grants: Bill and Melinda Gates Foundation OPP1152812,OPP1066118, OPP1136759, OPP1138727, and OPP1144149, and Advanced Imaging and Tissue Analysis Core of the Washington University Digestive Diseases Research Core Center P30DK052574.

## Data availability

Data described in the manuscript, code book, and analytic code will be made available upon request to the corresponding author pending application and approval.

## Figures and Tables

**Table:** 

Surname	Name	Full Affiliation	Email
Ahmed	Kumail	Department of Paediatrics and Child Health, Aga Khan University, Karachi, Pakistan	kumail.ahmed@aku.edu
Ahmed	Sheraz	Department of Paediatrics and Child Health, Aga Khan University, Karachi, Pakistan	sheraz.ahmed@aku.edu
Alam	Md Ashraful	Nutrition Research Division, International Centre for Diarrhoeal Disease Research, Bangladesh, Dhaka, Bangladesh	mashraful@icddrb.org
Amadi	Beatrice	Tropical Gastroenterology and Nutrition Group, University of Zambia School of Medicine, Lusaka, Zambia	beatriceamadi@ymail.com
Banda	Rosemary	Tropical Gastroenterology and Nutrition Group, University of Zambia School of Medicine, Lusaka, Zambia	rosemarybanda2@gmail.com
Dars	Shareef	Department of Paediatrics and Child Health, Aga Khan University, Karachi, Pakistan	shareef.dars@aku.edu
Das	Subhasish	Nutrition Research Division, International Centre for Diarrhoeal Disease Research, Bangladesh, Dhaka, Bangladesh	subhasish.das@icddrb.org
Denson	Lee A	Division of Pediatric Gastroenterology, Hepatology, and Nutrition, Cincinnati Children's Hospital Medical Center, Cincinnati, OH, USA	Lee.Denson@cchmc.org
Hossain	Md Shabab	Nutrition Research Division, International Centre for Diarrhoeal Disease Research, Bangladesh, Dhaka, Bangladesh	dr.shabab@icddrb.org
Hotwani	Aneeta	Department of Paediatrics and Child Health, Aga Khan University, Karachi, Pakistan	aneeta.hotwani@aku.edu
Iqbal	Junaid	Department of Paediatrics and Child Health, Aga Khan University, Karachi, Pakistan	junaid.iqbal@aku.edu
Iqbal	Najeeha Talat	Department of Biological and Biomedical Sciences, Aga Khan University, Karachi, Pakistan	najeeha.iqbal@aku.edu
Jakhro	Sadaf	Department of Paediatrics and Child Health, Aga Khan University, Karachi, Pakistan	sadaf.jakhro@aku.edu
Kabir	Furqan	Department of Paediatrics and Child Health, Aga Khan University, Karachi, Pakistan	furqan.kabir@aku.edu
Kazhila	Lydia	Tropical Gastroenterology and Nutrition Group, University of Zambia School of Medicine, Lusaka, Zambia	lydia@tropgan.net
Liu	Ta-Chiang	Department of Pathology and Immunology, Washington University School of Medicine, St. Louis, MO, USA	ta-chiang.liu@wustl.edu
Mann	Barbara J	Department of Medicine, University of Virginia School of Medicine, Charlottesville, Virginia, USA	bjm2r@virginia.edu
Memon	Waheeda	Department of Paediatrics and Child Health, Aga Khan University, Karachi, Pakistan	waheeda.memon@aku.edu
Moskaluk	Christopher A	Department of Pathology, University of Virginia School of Medicine, Charlottesville, VA, USA	cam5p@virginia.edu
Qureshi	Abdul Khalique	Department of Paediatrics and Child Health, Aga Khan University, Karachi, Pakistan	abdul.qureshi@aku.edu
Ragahavan	Shyam S	Department of Pathology, University of Virginia School of Medicine, Charlottesville, VA, USA	SR7WQ@hscmail.mcc.virginia.edu
Rahman	Masudur	Department of Gastroenterology, Sheikh Russel National Gastroliver Institute and Hospital, Dhaka, Bangladesh	drmasud47@yahoo.com
Rahman	Najeeb	Department of Paediatrics and Child Health, Aga Khan University, Karachi, Pakistan	najeeb.rahman@aku.edu
Sadiq	Kamran	Department of Paediatrics and Child Health, Aga Khan University, Karachi, Pakistan	kamran.sadiq@aku.edu
Sarker	Shafiqul Alam	Nutrition Research Division, International Centre for Diarrhoeal Disease Research, Bangladesh, Dhaka, Bangladesh	sasarker@icddrb.org
Sullivan	Peter B	Department of Paediatrics, Children's Hospital, University of Oxford, Oxford, UK	peter.sullivan@paediatrics.ox.ac.uk
Tarr	Phillip I	Department of Pediatrics, Washington University School of Medicine, St. Louis, MO, USA	tarr@wustl.edu
Tearney	Guillermo J	Department of Pathology, Harvard Medical School, Boston, MA, USA	gtearney@partners.org
Umrani	Fayaz	Department of Paediatrics and Child Health, Aga Khan University, Karachi, Pakistan	fayaz.umrani@aku.edu
Yilmaz	Omer H	Department of Pathology, Massachusetts General Hospital, Boston, MA, USA	oyilmaz@mgh.harvard.edu
