# Supplementary material for: Anthropometry relationship with duodenal histologic features of children with environmental enteric dysfunction: a multicenter cross-sectional study
Source: Am J Clin Nutr. 2024 Sep 17;120(Suppl 1):S65–72. doi: 10.1016/j.ajcnut.2024.02.027 (PMC13168959; doi:10.1016/j.ajcnut.2024.02.027)
Supplement: Supplementary file 1 [file mmc1.zip › ajcnut_469_ZEHRAJ~1_mmc1.DOC]

Supplementary Table 1. Description of histology parameter scoring.

| **Goblet cell density depletion^1^** | **0:** Normal goblet cell density (at least 1 goblet cell per 20 enterocytes) in all evaluable mucosal epithelial layer | **1:** Decreased goblet cells (<1/20 enterocytes) in 1-25% of evaluable mucosal epithelium | **2:** Decreased goblet cells (<1/20 enterocytes) in 26-50% of evaluable mucosal epithelium | **3:** Decreased goblet cells (<1/20 enterocytes) in 51-75% of evaluable mucosal epithelium | **4:** Decreased goblet cells (<1/20 enterocytes) in 76-100% of evaluable mucosal epithelium | **NS:** Not scorable |
| --- | --- | --- | --- | --- | --- | --- |
| **Intra-**  **epithelial lymphocytes^1^** | **0:** No areas observed with epithelial/lymphocyte ratio >20% | **1:** Lymphocyte/ epithelial ratio >20%, but <50%, in less than 50% of mucosa | **2:** Lymphocyte/ epithelial ratio >20%, but <50%, in greater than 50% of mucosa | **3:** Lymphocyte/ epithelial ratio >50% in less than 50% of mucosa | **4:** Lymphocyte/ epithelial ratio >50% in greater than 50% of mucosa | **NS:** Not scorable |
| **Intramucosal Brunner’s glands^1^** | **0:** None observed | **1:** One or two foci of intramucosal Brunner glands, none involving more than 5 crypt bases | **2:** 3-5 foci of intramucosal Brunner glands, none involving more than 5 crypt bases | **3:** > 5 foci, or any area of intramucosal Brunner glands involving >5 crypt bases |  | **NS:** Not scorable |
| **Paneth cell density depletion^1^** | **0:** >5 Paneth cells/ crypt base, on average | **1:** 2-4 Paneth cells/ crypt base, on average | **2:** <2 Paneth cell/crypt base, involving <50% of crypt bases | **3:** <2 Paneth cell/crypt, involving >50% of crypt bases |  | **NS:** Not scorable |
| **Villus architecture^1^** | **0:** Majority of villi are >3 crypt lengths long | **1:** Villi are < 3 but > 1 crypt lengths long, with abnormality involving ≤ 50% of mucosa | **2:** Villi are < 3 but > 1 crypt lengths long, with abnormality involving > 50% of mucosa | **3:** Villi absent, or <1 crypt length long, with abnormality involving ≤ 50% of mucosa | **4:** Villi absent, or <1 crypt length long, with abnormality involving > 50% of mucosa | **NS:** Not scorable |
| **Chronic Inflammation** | **0:** No qualitative increase in mononuclear inflammatory cells in lamina propria. Majority of villus bases contain <3 MIC across, on average | **1:** Increased MIC, based on villus base displaying 3-5 MIC across, on average | **2:** Increased MIC, based on villus base displaying 6-10 MIC across, on average | **3:** Increased MIC, based on villus base displaying >10 lymphocytes on average |  | **NS:** Not scorable |
| **Enterocyte injury** | **0:** Majority of enterocytes (90%) show tall columnar morphology | **1:** Enterocytes show low columnar (<2:1 L:W ratio), cuboidal or flat morphology, in ≤ 50% of mucosa | **2:** Enterocytes show low columnar (<2:1 L:W ratio), cuboidal or flat morphology, in > 50% of mucosa | **3:** Any area of mucosal erosion/ulceration |  | **NS:** Not scorable factor |
| **Epithelial detachment** | **0:** Complete coverage of mucosal surface by epithelial cells | **1:** Surface epithelium missing or detached from <25% of mucosa | **2:** Surface epithelium missing or detached from 25-50% of mucosa | **3:** Surface epithelium missing or detached from 51-75% of mucosa | **4:** Surface epithelium missing or detached from >75% of mucosa | **NS:** Not scorable |

**^1^**These five histology parameters are included in the calculation of the Total Score Percent-5 (TSP-5). At least four of these five parameters must have a numeric score (i.e., not non-scorable) for a slide to have a TSP-5 calculated. The numerator of the TSP-5 is the sum of the score of the scorable parameters and the denominator is the sum of the possible maximum score of the scorable parameters.

Abbreviations: NS, not scorable; variable cannot be determined because of slide quality or other factor

Supplementary Table 2**.** Distribution of participants by underweight, stunting, wasting status

| Nutritional status | | | EED | | | Nondiagnostic | Celiac |
| --- | --- | --- | --- | --- | --- | --- | --- |
|  |  |  | SEEM | BEECH | BEED | CCHMC/UVA | CCHMC/UVA |
| Underweight | Wasted | Stunted | 32 (50.8%) | 5 (4.6%) | 11 (9.5%) |  |  |
|  |  | Not stunted | 5 (7.9%) | 1 (0.9%) | 3 (2.6%) | 1 (2.3%) |  |
|  | Not wasted | Stunted | 19 (30.2%) | 63 (58.3%) | 26 (22.4%) |  |  |
|  |  | Not stunted | 2 (3.2%) |  | 4 (3.4%) |  |  |
|  | N/A^1^ | Stunted |  |  |  | 1 (2.3%) |  |
|  |  | Not stunted |  |  |  | 1 (2.3%) |  |
| Not underweight | Wasted | Not stunted | 1 (1.6%) |  |  | 1 (2.3%) |  |
|  | Not wasted | Stunted | 1 (1.6%) | 38 (35.2%) | 28 (24.1%) |  |  |
|  |  | Not stunted | 3 (4.8%) | 1 (0.9%) | 44 (37.9%) | 8 (18.6%) | 5 (22.7%) |
|  | N/A^1^ | Not stunted |  |  |  | 31 (72.1%) | 17 (77.3%) |
| Total |  |  | 63 | 108 | 116 | 43 | 22 |

^1^WHZ can only be calculated for children <5 years. All EED cohorts included children <5 years. CCHMC celiac cohort included 4; UVA celiac included 1, CCHMC non-diagnostic included 10, and UVA non-diagnostic included 0.

Abbreviations: HAZ, height-for-age Z score; N/A, not available; WAZ, weight-for-age Z score; WHZ, weight-for-height Z score

Supplementary Table 3: Summary histology scores by Center and disease, reported as medians (interquartile range) (% not scorable)

| Variable and range of values | EED Centers | | | | US celiac disease | | | US nondiagnostic | | |
| --- | --- | --- | --- | --- | --- | --- | --- | --- | --- | --- |
|  | icddr,b  (BEED)  N=116 | AKU  (SEEM)  N=63 | UTH  (BEECH)  N=108 | Combined  N=287 | UVA  N=2 | CCHMC  N=20 | Combined  N=22 | UVA  N=15 | CCHMC N=28 | Combined  N=43 |
| Total Score Percent – Top 5  (0-100%) | 58.6  (47.2, 66.7) (32.8) | 44.5  (36.7, 52.4)  (0) | 54.4  (47.1, 59.8)  (3.7) | 52.8  (44.4, 60.6)  (14.6) | 55.5  (52.8, 58.3) (0) | 40.3  (30.6, 48.7) (0) | 44.5  (31.3, 50.0)  (0) | 11.1  (7.5, 21.2) (0) | 11.9  (7.2, 17.0)  (0) | 11.1  (7.2, 18.3)  (0) |
| Goblet Cell Depletion (0-4) | 2.0  (1.4, 2.5)  (0) | 1.0  (0.8, 1.3)  (0) | 1.5  (1.0, 2.0)  (1.9) | 1.5  (1.0, 2.0)  (0.7) | 0.4  (0.3, 0.4)  (0) | 0.5  (0.5, 1.0)  (0) | 0.5  (0.5, 1.0)  (0) | 0.3  (0.0, 0.5)  (0) | 0.0  (0.0, 0.3)  (0) | 0.0  (0.0, 0.5)  (0) |
| Intramucosal Brunner Glands (0-3) | 0.0  (0.0, 0.5)  (3.4) | 0.2  (0.0, 1.0)  (0) | 0.0  (0.0, 0.2)  (0.9) | 0.0  (0.0, 0.5)  ( 1.7) | 1.0  (0.5, 1.5)  (0) | 3.0  (3.0, 3.0)  (0) | 3.0  (3.0, 3.0)  (0) | 3.0  (1.1, 3.0) (0) | 2.4  (0.7, 3.0)  (0) | 3.0  (1.0, 3.0)  (0) |
| Intraepithelial Lymphocytes (0-4) | 1.5  (1.0, 2.0)  (0.9) | 1.7  (1.2, 2.5)  (0) | 1.0  (0.5, 1.5)  (0.9) | 1.5  (1.0, 2.0)  ( 0.7) | 2.5  (2.2, 2.8)  (0) | 3.0  (1.9, 3.1)  (0) | 3.0  (2.0, 3.0)  (0) | 0.3  (0.0, 0.5)  (0) | 0.5  (0.0, 0.7)  (0) | 0.3  (0.0, 0.5)  (0) |
| Paneth Cell Depletion (0-3) | 3.0  (2.0, 3.0)  (46.6) | 0.7  (0.5, 1.0)  (1.6) | 2.0  (1.0, 3.0)  (11.1) | 1.5  (1.0, 3.0)  (23.3) | 1.1  (0.9, 1.3)  (0) | 0.5  (0.4, 0.8)  (0) | 0.5  (0.5, 0.9)  (0) | 0.3  (0.1, 0.5)  (0) | 0.0  (0.0, 0.5)  (0) | 0.0  (0.0, 0.5)  (0) |
| Villus Architecture (0-4) | 2.0  (1.0, 3.0)  (38.8) | 2.1  (1.3, 3.0)  (9.5) | 2.5  (1.8, 3.3)  (6.5) | 2.0  (1.5, 3.0)  (20.2) | 4.0  (4.0, 4.0)  (0) | 3.2  (2.4, 4.0)  (0) | 3.8  (2.5, 4.0)  (0) | 0.5  (0.2, 0.8)  (20) | 0.3  (0.0, 0.5)  (3.6) | 0.5  (0.0, 0.5)  (9.3) |
| Chronic Inflammation (0-3) | 1.5  (1.0, 1.5)  (1.7) | 1.3  (1.0, 1.6)  (0) | 1.5  (1.2, 2.0)  (1.9) | 1.5  (1.0, 1.7)  ( 1.4) | 2.2  (2.1, 2.4)  (0) | 2.0  (1.5, 2.0)  (0) | 2.0  (1.6, 2.0)  (0) | 1.0  (0.6, 1.0)  (0) | 0.8  (0.4, 1.0)  (0) | 1.0  (0.5, 1.0)  (0) |
| Enterocyte Injury (0-3) | 0.3  (0.0, 0.5)  (0) | 0.3  (0.2, 0.5)  (0) | 0.2  (0.0, 0.5)  (0.9) | 0.3  (0.0, 0.5)  ( 0.3) | 1.1  (1.1, 1.2)  (0) | 0.8  (0.4, 1.1)  (0) | 1.0  (0.5, 1.2)  (0) | 0.3  (0.0, 0.4)  (0) | 0.0  (0.0, 0.1)  (0) | 0.0  (0.0, 0.3)  (0) |
| Epithelial Detachment (0-4) | 1.0  (0.5, 1.0)  (0) | 1.0  (0.8, 1.2)  (0) | 1.0  (0.7, 1.2)  (0) | 1.0  (0.7, 1.2)  (0) | 0.8  (0.6, 0.9)  (0) | 1.0  (0.9, 1.1)  (0) | 1.0  (0.8, 1.0)  (0) | 1.0  (1.0, 1.6)  (0) | 0.7  (0.5, 1.0)  (0) | 0.7  (0.6, 1.0)  (0) |

Supplementary Table 4A: Distribution of study participants into four categories based on WAZ scores

|  | WAZ categories | Statistic | EED | | | EED sites combined | Normal | Celiac |
| --- | --- | --- | --- | --- | --- | --- | --- | --- |
|  |  |  | AKU | UTH | icddr,b |  | CCHMC/UVA | CCHMC/UVA |
| Total score percent-5 (possible range 0-100) | (-6.6 to <-3) | Median | 46.8 | 54.4 | 53.4 | 50 | NA | NA |
|  |  | q25 | 35.6 | 49.8 | 47.9 | 38.9 | NA | NA |
|  |  | q75 | 54.2 | 57.8 | 66.2 | 56.8 | NA | NA |
|  |  | N | 34 | 12 | 6 | 52 | 0 | 0 |
|  | (-3 to <-2) | Median | 45.4 | 53.5 | 55.6 | 52.4 | 13.9 | NA |
|  |  | q25 | 39.5 | 47.2 | 45.5 | 44.4 | 12.5 | NA |
|  |  | q75 | 52.3 | 61.1 | 60.9 | 60 | 15 | NA |
|  |  | N | 24 | 57 | 27 | 108 | 3 | 0 |
|  | (-2 to <-1) | Median | 41 | 55.6 | 60.9 | 56.6 | 17.8 | 38.9 |
|  |  | q25 | 37.8 | 47 | 53.2 | 47.2 | 10.6 | 36.1 |
|  |  | q75 | 42.4 | 58.3 | 71.9 | 63.9 | 21.9 | 47.2 |
|  |  | N | 5 | 32 | 32 | 69 | 6 | 5 |
|  | (-1 to 6) | Median | NA | 30.6 | 52.8 | 51.4 | 8.85 | 47.2 |
|  |  | q25 | NA | 25 | 47.2 | 46.1 | 7.2 | 30.6 |
|  |  | q75 | NA | 47.2 | 67.2 | 66.8 | 18.2 | 52.8 |
|  |  | N | 0 | 3 | 13 | 16 | 34 | 17 |

Supplementary Table 4B: Distribution of study participants into four categories based on WLZ/WHZ scores

|  | WHZ categories | Statistic | EED | | | EED sites combined | Normal | Celiac |
| --- | --- | --- | --- | --- | --- | --- | --- | --- |
|  |  |  | AKU | UTH | icddr,b |  | CCHMC/UVA | CCHMC/UVA |
| Total score percent-5 (possible range 0-100) | (-6.6 to <-3) | Median | 46.4 | 50 | 47.2 | 46.8 | NA | NA |
|  |  | q25 | 36.8 | 45 | 47.2 | 39.2 | NA | NA |
|  |  | q75 | 52.5 | 55 | 47.2 | 53.1 | NA | NA |
|  |  | N | 11 | 2 | 1 | 14 | 0 | 0 |
|  | (-3 to <-2) | Median | 50 | 55 | 52.2 | 50 | 12.8 | NA |
|  |  | q25 | 39.5 | 45.8 | 41.3 | 39.3 | 11.9 | NA |
|  |  | q75 | 55.6 | 58.5 | 56.4 | 56.7 | 13.6 | NA |
|  |  | N | 27 | 4 | 10 | 41 | 2 | 0 |
|  | (-2 to <-1) | Median | 41.6 | 55.6 | 58.9 | 53.3 | 22.2 | NA |
|  |  | q25 | 35.5 | 49.6 | 47 | 44.4 | 22.2 | NA |
|  |  | q75 | 47.8 | 63.3 | 65.3 | 60.6 | 22.2 | NA |
|  |  | N | 20 | 37 | 31 | 88 | 1 | 0 |
|  | (-1 to 6) | Median | 44.4 | 52.8 | 61.1 | 54.3 | 15 | 48.3 |
|  |  | q25 | 42.4 | 46.4 | 49.7 | 47.2 | 8.3 | 38.9 |
|  |  | q75 | 50 | 58.3 | 71.9 | 61.1 | 20.2 | 52.8 |
|  |  | N | 5 | 61 | 36 | 102 | 7 | 5 |
